# A novel *PIEZO1* mutation in a patient with dehydrated hereditary stomatocytosis: a case report and a brief review of literature

**DOI:** 10.1186/s13052-020-00864-x

**Published:** 2020-07-23

**Authors:** Daniele Zama, Giulia Giulietti, Edoardo Muratore, Immacolata Andolfo, Roberta Russo, Achille Iolascon, Andrea Pession

**Affiliations:** 1 Department of Pediatrics, “Lalla Seràgnoli,” Hematology-Oncology Unit, Sant’Orsola-Malpighi Hospital, University of Bologna, Via Massarenti 11, 40137 Bologna, Italy; 2 Department of Molecular Medicine and Medical Biotechnologies, “Federico II” University of Naples, Naples, Italy; 3 CEINGE, Biotecnologie Avanzate, Naples, Italy

**Keywords:** Hemolytic anemia, Dehydrated hereditary stomatocytosis, Mean corpuscular hemoglobin concentration (MCHC), Genetic disease, Next generation sequencing (NGS)

## Abstract

**Background:**

Dehydrated hereditary stomatocytosis (DHS) or hereditary xerocytosis is a rare, autosomal dominant hemolytic anemia characterized by macrocytosis, presence of stomatocytes and dehydration of red blood cells (RBCs). The dehydration is caused by a defect in cellular cation content. The most frequent expression of the pathology is hemolytic well-compensated anemia with high reticulocyte count, a tendency to macrocytosis, increased mean corpuscular hemoglobin concentration (MCHC) and mild jaundice. We here describe a new mutation of *PIEZO1* gene, the most frequent mutated gene in DHS, in a family affected by hereditary hemolytic anemia.

**Case presentation:**

We describe the case of a 12-years-old girl with well-compensated chronic hemolysis, increased MCHC and a father who had the same hematological characteristics. After excluding secondary causes of chronic hemolysis and enzymatic defects of the RBCs, microscopic observation of the peripheral blood smear, tests of RBC lysis, ektacytometry, SDS-PAGE and in last instance genetic analysis has been performed. This complex diagnostic workup identified a new variant in the *PIEZO1* gene, never described in literature, causative of DHS. This pathogenetic variant was also detected in the father.

**Conclusions:**

This case report highlights the importance of a correct and exhaustive diagnostic-workup in patients with clinical suspicious for hemolytic anemia in order to make a differential diagnosis. This is relevant for the management of these patients because splenectomy is contraindicated in DHS due to high thrombotic risk.

## Background

Dehydrated hereditary stomatocytosis (DHS) or hereditary xerocytosis (OMIM#194380) is a rare, autosomal dominant congenital hemolytic anemia, characterized by large and dehydrated red blood cells (RBCs) [1]. DHS is the most common type among the large group of hereditary stomatocytosis. It is 10–20 times less frequent than hereditary spherocytosis, and shares with it some common features, leading to possible missdiagnosis [1].

It is caused by an alteration of the RBC membrane permeability to the monovalent cations Na + and K+, with a consequent alteration of the intracellular cationic content, cell dehydration, and modifications of cell volume. The main causative gene is *PIEZO1* [2], localized at 16q23–24. PIEZO1 (OMIM#194380) encodes for a mechanoreceptor, that is a cation channel activated by various types of mechanical stimuli, and that functions as a biological pressure sensor in both vertebrates and invertebrates. It is high expressed at plasma membrane of RBCs in which it regulates the cell volume [3–6]. The identified mutations are mostly missense and mainly located in the highly conserved C-terminus of the protein, recently described to form the pore of the channel [7]. Several electrophysiology studies demonstrated that the mutations cause a gain-of-function phenotype with delayed inactivation of the channel [2, 8, 9], explaining the increased cation permeability that leads to erythrocyte dehydration. Mutations of *KCNN4* (OMIM#616689), encoding the Gardos channel, a widely expressed Ca2 + −dependent K+ channel of intermediate conductance that mediates the major K+ conductance of erythrocytes have also been described recently in literature as causative of DHS2 [10, 11].

The clinical phenotype of DHS ranges from asymptomatic to severe forms, with massive hemolysis. Sign and symptoms comprise mild to severe anemia, jaundice, pallor, fatigue, splenomegaly, gallstones, and severe iron overload [1, 7, 9].

DHS patients generally show hemolytic well-compensated anemia, with a high reticulocyte count, a tendency to macrocytosis and mild jaundice. The main characteristic of RBCs is cell dehydration caused by the loss of the cation content, with a subsequent increase of MCHC (> 36 g/dL). At blood smear, the stomatocytes, erythrocytes with a characteristic central mouth-shaped spot [2], are quite rare, which often makes diagnosis difficult. Osmotic gradient ektacytometry is a useful and often critical examination to diagnose this condition. It shows a leftward shift of the osmolarity curve due to the presence of dehydrated erythrocytes [﻿12﻿].

DHS can be classified into a non-syndromic form, with only hematological involvement, and syndromic form, also called pleiotropic syndrome, characterized by anemia with pseudohyperkalemia and/or pre/peri-natal edema [2, 13, 14]. DHS is often undiagnosed or misdiagnosed with other conditions, especially hereditary spherocytosis.

## Case presentation

The proband was a 12-years-old girl presenting with chronic compensated hemolysis, Gilbert syndrome, and recurrent abdominal pain. The blood count showed: hemoglobin 13 g/dL, red blood cell counts 4.020.000/mm^3^, Mean Corpuscular Volume (MCV) 90.3 fL, Mean Content Hemoglobin (MCH) 32.1 pg, mean corpuscular hemoglobin concentration (MCHC) 38.3 g/dL, reticulocytosis, and indirect hyperbilirubinemia. The complete blood work-up is shown in Table 1. The ultrasound of the abdomen did not show gallstones, hepatomegaly and splenomegaly.

**Table 1:** Complete blood work-up of the patient

	Value	Reference Range
Red blood cell count (n/mmc)	4.020.000	4.200.000–6.100.000
Hemoglobin (g/dl)	13	12–18
Hematocrit (%)	36,3	37–52
Mean Corpuscolar Volume (fL)	90,3	80–99
Mean Corpuscolar Hemoglobin (pg)	32,1	27–31
Mean Corpuscolar Hemoglobin Concentration (g/dL)	38,3	33–37
Red-Cell distribution Widht (%)	12,9	11,5-14,5
Reticulocyte count (/mmc)	178.000	22.000–139.000
Reticulocyte count % (%)	4,43	0,5-2,5
Mean Corpuscolar Hemoglobin Concentration of Reticulocyte (g/dl)	32,9	23–29
Mean Corpuscolar Hemoglobin of Reticulocyte (pg)	33,7	25–30
Erythropoietin (mU/ml)	33,8	2,6-18,5
Total Bilirubin (mg/dl)	8,58	< 1,20
Direct Bilirubin (mg/dl)	0,72	< 0,30
Indirect Bilirubin (mg/dl)	7,86	< 0,90
Haptoglobin (mg/dl)	< 30	30–200
Lactate Dehydrogenase (U/l)	138	110–295
Hemoglobin A2 (%)	3,1	1,8-3,2
Hemoglobin F (%)	4	< 1
Serum iron level (mcg/dl)	193	60–18
Transferrin (mg/dl)	259	200–360
Ferritin (ng/dl)	43	11–306

**Fig. 1:**
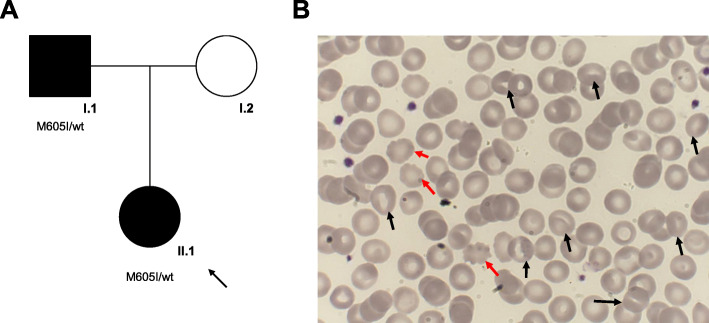
**a** The family tree comprising the proband (black arrow), the affected father and the unaffected mother. **b** Morphological examination of peripheral blood showing typical stomatocytes (black arrows) and acanthocytes (red arrows)

The patient was a single-born from non-consanguineous Italian parents (Fig. 1a). The remote case history reported neonatal jaundice treated with phototherapy. The family anamnesis revealed that the father presented jaundice during his childhood, recurrent episodes of abdominal pain resolved after splenectomy (performed at the age of 23 years-old) and gallstones for which he had undergone a cholecystectomy. The paternal uncle also had gallbladder stones. To investigate the cause of hemolysis in our patient, laboratory investigations were carried out. The peripheral blood smear showed anisocytosis with the presence of red blood cells with specific shape: stomatocytes (5%), ovalocytes (4%), schistocytes (3%) and rare spherocytes (Fig. 1b). The direct antiglobulin test (DAT) was negative, thus excluding an autoimmune origin of the hemolysis. Structural hemoglobin alterations have also been excluded performing electrophoresis and molecular assessment of the genes encoding beta and alpha-globin chains. The activity of red blood cell metabolism enzymes (Hexokinase, Glucose-6-phosphate isomerase, 6-Phosphofruttochinase, Glyceraldehyde phosphate dehydrogenase, Phosphoglycerate kinase, Pyruvate kinase, Glucose 6 phosphate dehydrogenase, Adenylate 6-Phosphogluconate dehydrogenase kinase) was normal.

The family history showed an autosomal dominant inheritance of the condition. To investigate the possible presence of a spherocytosis condition, the most frequent erythrocyte structural defect, a combination of tests was performed. First, the eosin-5′-maleimide binding test (EMA test), a cytometric analysis in which a fluoresceinated (eosin-5′-maleimide) substance binds to the plasma membrane proteins of red blood cells, mainly to the band 3 protein [1, 9]. The average fluorescence of RBCs with EMA staining in patients with spherocytosis is lower than that of control RBCs due to the decrease in the number of target proteins. In our case, this examination was in the normal range with a value of 12% (normal test value> 11%). The other complementary investigations carried out were the osmotic resistance tests such as the glycerol lysis test (AGLT50) and the Pink test. These tests were normal in our patient. Furthermore, quantitative analysis of membrane proteins was carried out using Sodium Dodecyl Sulphate-PolyAcrylamide Gel Electrophoresis (SDS-PAGE), and resulted not altered. We also performed the ektacytometry that evaluates the erythrocyte deformability by subjecting them to an increasing osmotic gradient with constant shear stress [12]. Ektacytometry showed a left shift of the osmolarity curve suggestive of DHS. In agreement with the clinical suspicion, genetic testing was carried out both in the patient and in the parents by a targeted-NGS custom panel composed of 86 causative genes of hereditary anemias. This panel is an updated version of a similar previously published one [15]. We found in both subjects the missense variant c.1815G > A, p.Met605Ile in *PIEZO1* gene (NM_001142864, CCDS54058) in heterozygous state. According to the guidelines of American College of Medical Genetics and Genomics (ACMG), we evaluated the pathogenicity of this variant by gathering evidence from various sources: population data, computational and predictive data, functional data, and segregation data [16]. First, the variant segregated in the affected father (Fig. 1a). Currently, this variant is annotated neither in population databases nor in databases of known variant. Moreover, it is predicted by several tools as probably damaging (MutationTaster score 0.999 Disease causing; FATHMM Score − 1.59; LRT prediction Deleterious; PolyPhen2 score HumVar 0.968 Probably damaging; PROVEAN Score-2.95 Damaging).

## Discussion and conclusions

**Fig. 2:**
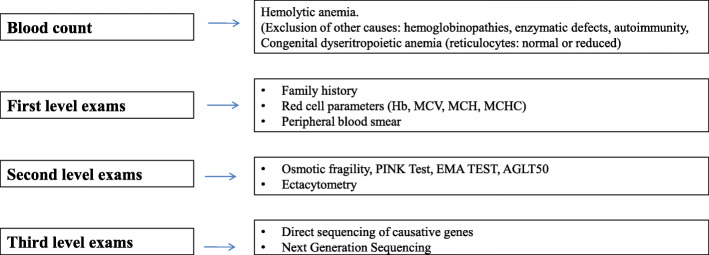
Laboratory tests that guide the differential diagnosis [7]

Our case demonstrates the complexity of differential diagnosis of hemolytic anemias in pediatric patients. A correct diagnostic workup should consider autoimmunity, enzymatic defects of the RBCs and erythrocyte membrane alterations, including, spherocytosis, elliptocytosis, ovalocytosis of South Asia, pyropoikilocytosis and stomatocytosis. Secondary causes, such as liver disease, must be also ruled oute [17] (Fig. 2).

The first line investigation includes erythrocyte parameters (in particular Hb, MCV, MCH, and MHCH), observation of the peripheral blood smear, and evaluation of the family history. The second line includes EMA test, osmotic fragility test, AGLT-50 test, and the pink test, that can address the diagnosis. It is also crucial to carry out ektacytometry analysis which shows characteristics deformation of the red blood cells, allowing to guide the diagnosis towards a precise pathology of the erythrocyte membrane. The third diagnostic line is represented by the sequencing of the candidate causative genes or the contemporary sequencing of a panel of genes by Next Generation Sequencing (NGS) technique, that has allowed the identification of numerous causative mutations associated with defined morphological pictures, with reduced times and costs [15, 18, 19].

Herein, we identified a novel *PIEZO1* variant that is located in non-pore domain of the PIEZO1 mechanoreceptor, responsible for the mechanosensitive properties of the channel [7]. Our recent genotype-phenotype correlation analysis on 29 PIEZO1-patients demonstrated that most of severely affected patients carried mutations in the pore domain, responsible of the ion passage, while patients showing a less severe phenotype carried mutations in the nonpore domain [7]. In accordance to this finding, the patients here described, both proband and father, showed a mild phenotype and the absence of iron overload [20].

Regarding treatment, for stomatocytosis, as for other RBC membrane pathologies, therapy is phototherapy/exanguino-transfusion in newborns suffering from this condition. Transfusions may also be necessary at any time of life due to aplastic and hemolytic crises.

It is particularly important to diagnose this pathology and to distinguish it from spherocytosis, because of the clinical consequences (Table 2). Indeed, patients with DHS have a higher risk of thromboembolic complications (portal thrombosis and pulmonary hypertension) [21] after splenectomy than in spherocytosis, due to an increased number of stomatocytes, not destroyed in the spleen, present in systemic circulation. Splenectomy is thus contraindicated in this condition. In addition, in patients with DHS iron metabolism should be regularly checked for the risk of developing sever hepatic iron overload [20] (Table 2).

**Table 2:** DHS and spherocytosis in comparison

	DHS	Spherocytosis
**MCHC**	Increased	Increased
**RDW**	Increased	Increased
**MCV**	Tendency to macrocytosis	Normal or slightly reduced
**Peripheral blood smear**	Stomatocytes	Spherocytes
**Splenomegaly**	mild / moderate	Variable from mild to severe
**EMA test and RBCs lysis tests**	Normal/Increased	Decreased
**Ectacytometry**	Normal EImax and left shift of Omin and OHyper due to the presence of dehydrated red cells.	Reduction of EImax, shift to the right of Omin point (reduced surface / volume ratio) and shift to the left of Ohyper point (increased dehydration of the red cells).
**Causative genes**	PIEZO1/ KCNN4	ANK1/ SPTB/ SPTA1/ SLC4A1/ EPB42

In conclusion, our case report shows that the diagnosis of DHS can be difficult and there can be a diagnostic delay if it is not properly suspected. Thus, we advise the use of a structured and exhaustive diagnostic workup. The application of a precise workflow led us to discover a new causative variant not yet described, highlighting the importance of a complete genetic workup in this category of patients.

## Data Availability

Data sharing not applicable to this article as no datasets were generated or analysed during the current study. The data used and/or analyzed during the writing of this manuscript are available from the corresponding Author on reasonable request.

## Abbreviations

ACMG: American College of Medical Genetics and Genomics
AGLT50: Acidified glycerol lysis test
DHS: Hereditary dehydrated stomatocytosis
EMA test: Eosin-5′-maleimide binding test
MCH: Mean Content Hemoglobin
MCHC: Mean corpuscular hemoglobin concentration
MCV): Mean corpuscular volume
RBC: Red blood cell
RDW: Red blood cells distribution width
SDS-PAGE: Sodium Dodecyl Sulphate - PolyAcrylamide Gel Electrophoresis
